# Vertical and bevel-structured SiC etching techniques incorporating different gas mixture plasmas for various microelectronic applications

**DOI:** 10.1038/s41598-017-04389-y

**Published:** 2017-06-20

**Authors:** Ho-Kun Sung, Tian Qiang, Zhao Yao, Yang Li, Qun Wu, Hee-Kwan Lee, Bum-Doo Park, Woong-Sun Lim, Kyung-Ho Park, Cong Wang

**Affiliations:** 1 Department of Electronic Engineering, Kwangwoon University, 20 Gwangun-Ro, Nowon-gu, Seoul, 139701 Republic of Korea; 2 Department of Microwave Engineering, Harbin Institute of Technology, Harbin, 150001 China; 3 Korea Advanced Nano Fab Center (KANC), 109 Gwanggyo-Ro, Yeongtong-gu, Suwon-si, Gyeonggi-do 443-270 Republic of Korea; 4 College of Electronic and Information Engineering, Qingdao University, Qingdao, 266071 China; 5 School of Information Science and Engineering, University of Jinan, Jinan, 250022 China

## Abstract

This study presents a detailed fabrication method, together with validation, discussion, and analysis, for state-of-the-art silicon carbide (SiC) etching of vertical and bevelled structures by using inductively coupled plasma reactive ion etching (ICP-RIE) for microelectronic applications. Applying different gas mixtures, a maximum bevel angle of 87° (almost vertical), large-angle bevels ranging from 40° to 80°, and small-angel bevels ranging from 7° to 17° were achieved separately using distinct gas mixtures at different ratios. We found that SF_6_ with additive O_2_ was effective for vertical etching, with a best etching rate of 3050 Å/min. As for the large-angle bevel structures, BCl_3_ + N_2_ gas mixtures show better characteristics, exhibiting a controllable and large etching angle range from 40° to 80° through the adjustment of the mixture ratio. Additionally, a Cl_2_ + O_2_ mixture at different ratios is applied to achieve a small-angel bevels ranging from 7° to 17°. A minimum bevel angel of approximately 7° was achieved under the specific volume of 2.4 sccm Cl_2_ and 3.6 sccm O_2_. These results can be used to improve performance in various microelectronic applications including MMIC via holes, PIN diodes, Schottky diodes, JFETs’ bevel mesa, and avalanche photodiode fabrication.

## Introduction

Silicon carbide (SiC) is a wide bandgap compound semiconductor with excellent thermal conductivity, high electric breakdown voltage, and high-temperature stability, making it a good material for high-power, high-frequency, or high-temperature electronic devices, such as Schottky diodes, field effect transistors (FETs), and high-efficiency light-emitting diodes^1–5^. However, SiC’s strong internal bonding energy results in a high chemical resistance, a property which eventually restricts the etching rate of the mask^6^, and its hardness (H = 9^+^ on the Mohs scale) makes chemical etching in traditional solutions difficult^7^. Because of its properties, SiC can also be used extensively for growth of an epitaxial layer of gallium nitride (GaN), used to form high-quality substrates for high-power monolithic microwave integrated circuit (MMIC) devices such as power amplifiers, low-noise amplifiers, and mixers^8–11^. This is especially convenient as SiC is susceptible to dry etching, producing SiC-based MMIC devices through backside vertical via-hole etching.

The proposed fabrication technology provides a basis for the future development of a wide variety of SiC-based devices. Various dry etching techniques have been used both to address the etching rate issue and ensure excellent selectivity^12–14^. Among the various dry etching techniques hitherto applied, inductively coupled plasma reactive ion etching (ICP-RIE) is the most widely adopted technique featuring a damage-free, highly anisotropic and selective, with a high etching rate. It also allows independent adjustment of the gas mixture and the flow rate^15–18^.

A variety of fluorine-, chlorine-, and bromine-based plasma chemistries—including NF_3_, NF_3_/O_2_, SF_6_/O_2_, SF_6_/He, SF_6_/O_2_/Ar, ICl, IBr, Cl_2_/Ar, and BCl_3_/Ar^14, 17–20^—have been studied for SiC etching. Among them, fluorine-based chemistries are the most effective gases when compared to other mixtures in terms of their ease of implementation and etching rate. In general, SiC dry etching is conducted using fluorine radicals as a primary source, followed by additive gases such as O_2_, Ar, N_2_, and H_2_ as a secondary source—to control and enhance the etching process. The highest etching rate can be achieved with both SF_6_ and NF_3_, because of their rapid dissociation in plasma; however, SF_6_ is the preferred feed gas based on cost and safety considerations^20^.

Recently, researchers have put plenty of efforts into developing various microelectronic devices based on the bevel structure. Large-angle bevel structure is commonly used in the application of PIN diodes, Schottky diodes, static induction transistors (SITs), and junction FETs (JFETs). To maintain the high performance and reliability of these devices, a smooth surface and accurate bevel angle should be guaranteed so that the issue of electric field concentration can be efficiently solved^21^. Several SiC diodes, transistors, and switches with high breakdown voltages (greater than 10 kV) have been reported, using a mesa shape with junction termination extensions formed by a notably large-angle bevel structure for SiC etching, which contributes to improve the breakdown voltage by alleviating the electric field crowding at the device edges^22^. As for the small-angle bevel, it plays an important role in the application of avalanche photodiodes (APD). Particular concern has been focus on the mesa structure with bevel sidewalls instead of vertical sidewalls. It can be referred that bevelling the sidewalls suppresses edge breakdown^23^. The effect of bevelled sidewalls is to increase the depletion width at the surface of the device, and therefore improve the breakdown voltage of the APD.

The use of the conventional SF_6_ + O_2_ gas mixture for bevel etch of the SiC material is limited by the difficulties in controlling both the mixture ratio and the RF power^24^. Accordingly, chlorine-based gas mixtures are carried out and have become notably promising for obtaining a smooth surface on the epitaxial layer. Most importantly, an independently controls of system parameters can be realized^25^.

In this work, the vertical etching of SiC using SF_6_/O_2_ plasma material with different ratios have been studied, resulting in an improved etch rate of 3050 Å/min. We have also found Ni to be a robust etch mask material, allowing a high selectivity of 100:1. Bevel structures with a large angle ranging from 40**°** to 80**°** were studied particularly under different gas mixtures, including BCl_3_/Cl_2_ and BCl_3_/N_2_, in which BCl_3_/N_2_ shows the best results in terms of obtaining controllable angles through adjusting the ratio of BCl_3_ with (BCl_3_ + N_2_). Large-angle bevels in SiC etching are significant for the growth of the epitaxial layer of AlGaN/GaN/SiC diodes, transistors, and switches. According to a preferred embodiment of the concept, an angle of approximately 60**°** optimizes the performance of the epitaxial layer. For other applications, smaller angles of approximately 40**°** are preferable, as they are less prone to peripheral breakdown^26^.

Small-angle bevel often encounters problems with early edge breakdown, as a locally enhanced electric field occurs on the etched junction surface at the active region of APDs during the mesa etching^27^. The formation of a small (~7**°**) SiC sidewall bevel structure is favoured to prevent such a premature edge breakdown^3^. Small-angle bevel structures (from 7**°** to 15**°**, in particular) are achieved using Cl_2_/O_2_ gas chemistry, which has been found in miniature, highly reliable, low power consumption, SiC-based APDs.

## Methods

This study combined various experiments to optimize the etching rate and selectivity on the 4H-SiC substrate. The performance of two different metal masks (Ni and Cr) was compared under a fixed SiC etching rate of 3050 Å/min. The more robust Ni mask, which allows for improved selectivity, was used as an etching mask in our other experiment. These masks were built to a thickness of 11 μm through e-beam evaporation at a deposition rate of 5 Å/sec and under a vacuum of 3.5 E^−6^ Torr.

Another experiment investigated the effects of different gas mixtures on the formation of various angles and bevel structures, ranging from as low as 7**°** (which can be effectively applied to APDs), 40**°**–80**°** (applicable for PIN diodes, Schottky diodes, and JFETs’ bevel mesa), and up to as high as 90**°** (interpreted as vertical etching, for application in MMICs through backside via-hole etching).

Further experimentation evaluated the impact of mixture rations (from 0 to 100%) in SF_6_/O_2_ gas on vertical bevel structure, using a fixed coil power of 2000 W and platen power of 200 W. The applied pressure was set up constantly at 20 mTorr, and the etching time was 5 h for all samples. Six samples were tested, with SF_6_ gas flow rates varying from 0 sccm to 25 sccm at 5 sccm intervals, while the O_2_ flow rate was inversely adjusted.

This study also developed an effective formation process for large-angle bevel-structured etching using BCl_3_ + N_2_ and BCl_3_ + Cl_2_ gas mixtures. Different conditions for SiC etching were studied using BCl_3_ with ratios ranging from 0 to 100%. Fixed process conditions (coil power = 900 W, platen power = 300 W, process pressure = 5 mTorr, and etching time = 30 min) were used for the ICP dry etching process with both BCl_3_ + N_2_ and BCl_3_ + Cl_2_. Furthermore, different mixtures of gases—including BCl_3_, BCl_3_ + Cl_2_, Cl_2_, and Cl_2_ + O_2_ at different ratios— were studied as a means to obtain small-angle bevel structures. In total, six different tests were conducted, including BCl_3_ alone at a volume of 6 sccm, BCl_3_ + Cl_2_ at volumes of 4.8 sccm BCl_3_ + 1.2 sccm Cl_2_, Cl_2_ alone at a volume of 6 sccm, and Cl_2 + _O_2_ at volumes of 4.8 sccm Cl_2 + _1.2 sccm O_2_, 3.6 sccm Cl_2 + _2.4 sccm O_2_, and 2.4 sccm Cl_2 + _3.6 sccm O_2_. A detailed summary of this experiment is illustrated in Table 1.

**Table 1:** Summarization of the detailed information of all experiments conducted in this work.

	Vertical Etching	Large-angle Bevel Etching		Small-angle Bevel Etching			
Gas Mixture	SF_6 + _O_2_	BCl_3 + _N_2_	BCl_3 + _Cl_2_	BCl_3_	BCl_3 + _Cl_2_	Cl_2_	Cl_2 + _O_2_
	Totally 25 sccm	Totally 40 sccm		Totally 6 sccm			
System Condition	Coil Power: 2000 W	Coil Power: 900 W		Coil Power: 0 W			
	Platen Power: 200 W	Platen Power: 300 W		Platen Power: 200 W			
	Applied Pressure: 20 mTorr	Applied Pressure: 5 mTorr		Applied Pressure: 3 mTorr			
	Etching Time: 5 h	Etching Time: 30 min		Etching Time: 30 min			

All of the experiment is based on 4H-SiC substrate, Ni mask is applied in the process of vertical etching and large-angle bevel etching, and AZ4620 photoresist is used as the etching mask for small-angle bevel etching.

## Results and Discussion

### Vertical Etching

**Figure 1:**
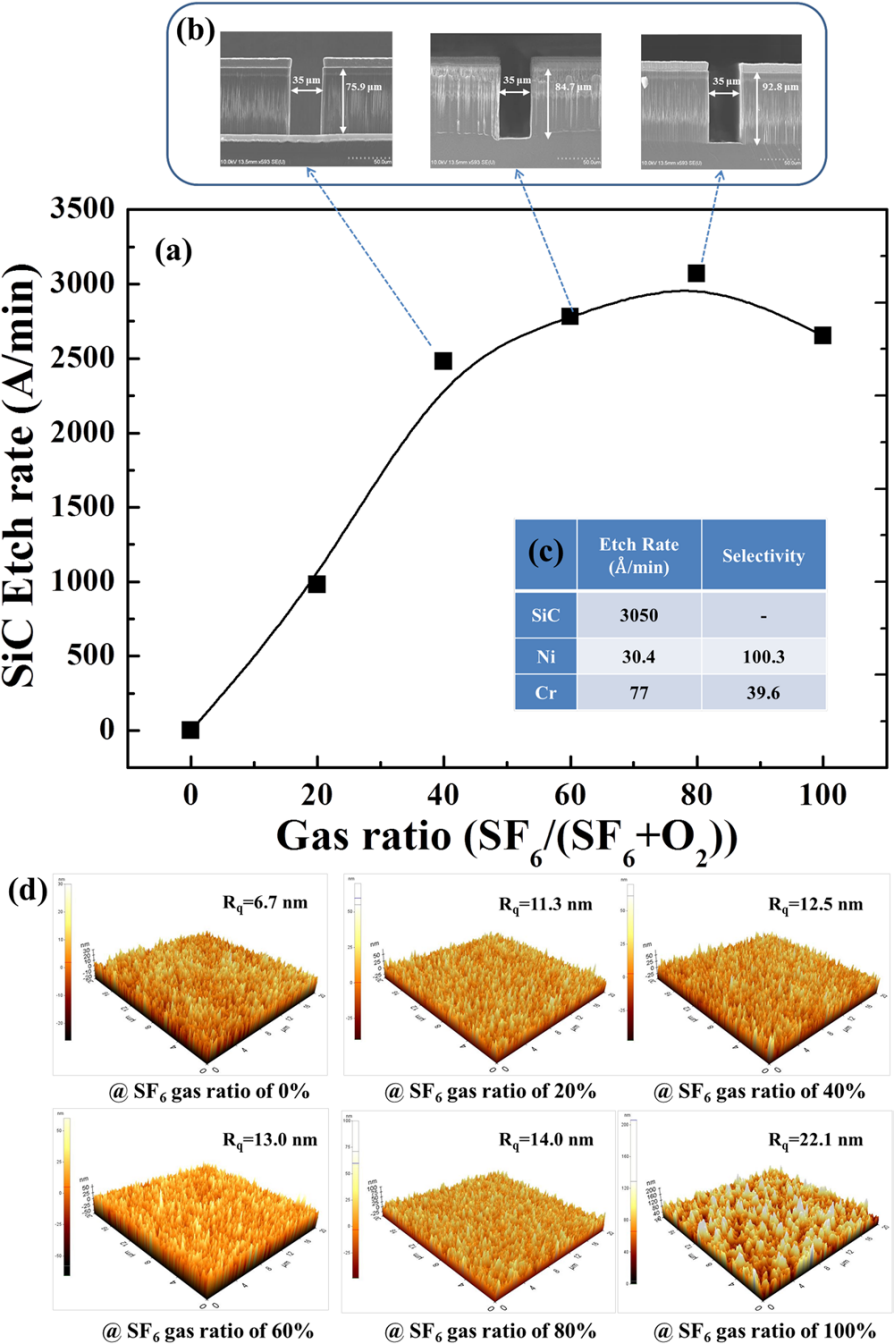
Etching and selectivity profile of the SF_6_ + O_2_ gas mixture. (**a**) Etching rate characteristics of the SF_6_ + O_2_ gas for different ratios. **(b)** Cross-sectional image of the etched SiC based on different gas ratio. **(c)** Etching rate and selectivity results for Ni and Cr metal masks, with a fixed SiC etching rate of 3050 Å/min. (**d**) AFM image of the etching surface at different ratios of SF_6_ + O_2_ gas.

Figure 1(a) demonstrates the changes in SiC etching rate when performed with different ratios of SF_6_ + O_2_. The highest SiC removal rate, 3050 Å/min, is achieved at the SF_6_ mixing ratio of 80%, whereas the etching rate tends to decrease for mixture ratios beyond 80%. The in reactive F^+^- ions, reactive gas dilution, removal efficiency of the etching products, decreased sulphur reaction efficiency, and competition from forming SiO_2_ likely all combine to result in the increase and eventual decline of the etching rate^28^. Although the addition of O_2_ to the SF_6_ plasma provides another pathway for volatilizing C in the forms CO, CO_2_, etc.—thereby increasing the SiC etching rate—it also produces SiO_2_ on the surface, which can limit the etching process. As a result of this competition, an optimum O_2_ ratio in the SF_6_/O_2_ gas mixture of around 20% is obtained.

The cross-sectional images of the etched SiC, based on the SF_6_ gas ratio of 40%, 60%, and 80%, are illustrated in Fig. 1(b) to show well-defined vertical etch structures, which verify the success of our proposed experiments and demonstrate the observed differences dependent on our proposed work from all of the etching conditions.

Figure 1(c) summarizes detailed measurement results of the Ni and Cr applied in this work. The etching rates observed when using Ni and Cr masks are 30 Å/min and 77 Å/min, respectively, when all other parameters are held constant to allow a SiC etching rate of 3050 Å/min. This discrepancy results in a selectivity of 100:1 for SiC:Ni and 40:1 for SiC:Cr. Because of this, the SF_6_-optimized etching conditions for SiC exhibit higher etching rates when Ni metal masks are used, approximately 3050 Å/min with a selectivity of 100:1. This was experimentally verified using a gas mixture of SF_6_ (20 sccm) + O_2_ (5 sccm). This preferential selectivity occurs because both the Ni and Cr metals react with fluoride gas, respectively forming NiF_2_ and CrF_3_, and the by-product NiF_2_ is more stable than CrF_3_ in air. As NiF_2_ is less volatile (the sublimation temperature of NiF_2_ is 1474 °C, whereas the anhydrous form of CrF_3_ sublimates at 1100–1200 °C), it causes less damage to the surface during etching, resulting in the higher selectivity^29, 30^. The slow etching rate observed with the Ni mask demonstrates that Ni is versatile enough be used as mask under various etching conditions. In contrast, Cr masks work only in fluorine-free environments, which minimize mask erosion.

**Figure 2:**
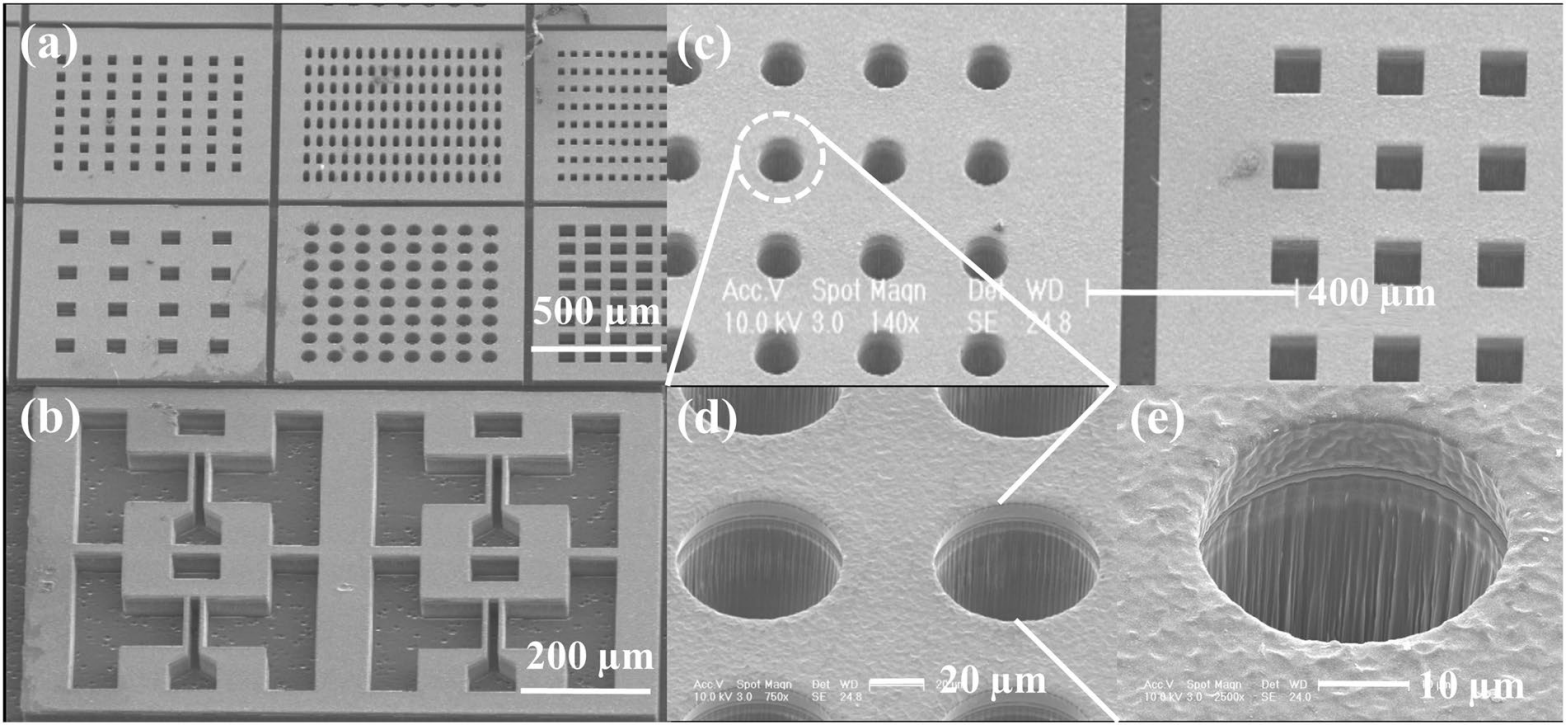
SEM images of various via-holes formed on a SiC substrate using optimized parameters for the ICP-RIE technique. (**a**) Mask opening widths of 35 µm, 25 µm, 20 µm, 100 µm, 70 µm, 70 µm (from the top left, row first). (**b**) Vertically etched SiC with a complicated pattern and a well-etched sidewall profile. (**c**) Mask opening width of 70 µm (circular) and 70 µm (square), from left to right. (**d**) Circular pattern enlarged five times. (**e**) Circular pattern enlarged ten times.

Atomic force microscopy (AFM) images are exhibited in Fig. 1(d), illustrating surface roughness after the following etching of gas mixtures with different ratios. It can be observed that surface roughness becomes less smooth with increasing of the SF_6_ proportion in SF_6_ + O_2_. As the ratio of SF_6_ increases, the surface roughness gradually deteriorates from 6.7 nm to 11.3 nm-14.0 nm before suddenly rebounding to 22.1 nm when the flow rate of SF_6_ is 25 sccm. The deterioration in the surface roughness occurs primarily because low-volatility reaction products, such as CF_x_ (CF_2_, CF_3_), are generated and scattered across the etching surface as a secondary mask. Generally, when more fluorine ions participate in the reaction, more products are created and etching surface becomes rougher. In addition, serious roughness deterioration occurs when the gas ration is over 80%, this is due to the fluorine atoms are of primary interest and they act as the main chemical reactants. The fraction of free surface increases rapidly, providing favourable conditions for both the physical sputtering etching and the chemical reaction. The optimized parameters used to achieve the 3050 Å/min etching rate with ICP dry etching are: *coil power* = 2000 W, *platen power* = 200 W, *processing mixture* = SF_6_ (20 sccm) + O_2_ (5 sccm), and *processing pressure* = 20 mTorr. Figure 2 shows a series of scanning electron microscopy (SEM) images of a 70-µm via-hole array formed on a SiC substrate, as obtained in this study.

### Large-angle Bevel Etching

Even though the SF_6_/O_2_ gas mixture can achieve a relative high etching rate, it is not the ideal solution for bevel etching; the SiC etching profile remains unchanged through variations in component ratio of the SF_6_/O_2_ gas mixture. This is because the most impactful species in SF_6_/O_2_ plasma processing is created when electrons collide with neutral gas molecules. These collisions result in dissociation (leading to radical formation), ionization (SF_5_^+^), and excitation, in accordance with the energy required for each process^31^. In an SF_6_/O_2_-SiC etching system, the O_2_ passivates the SiC surface with a SiO_2_ layer; subsequently, SF_5_^+^ ions etch these passivations and allow the F^+^ radicals to etch the SiC substrate beneath. Thus, anisotropic SiC etching is achieved.

**Figure 3:**
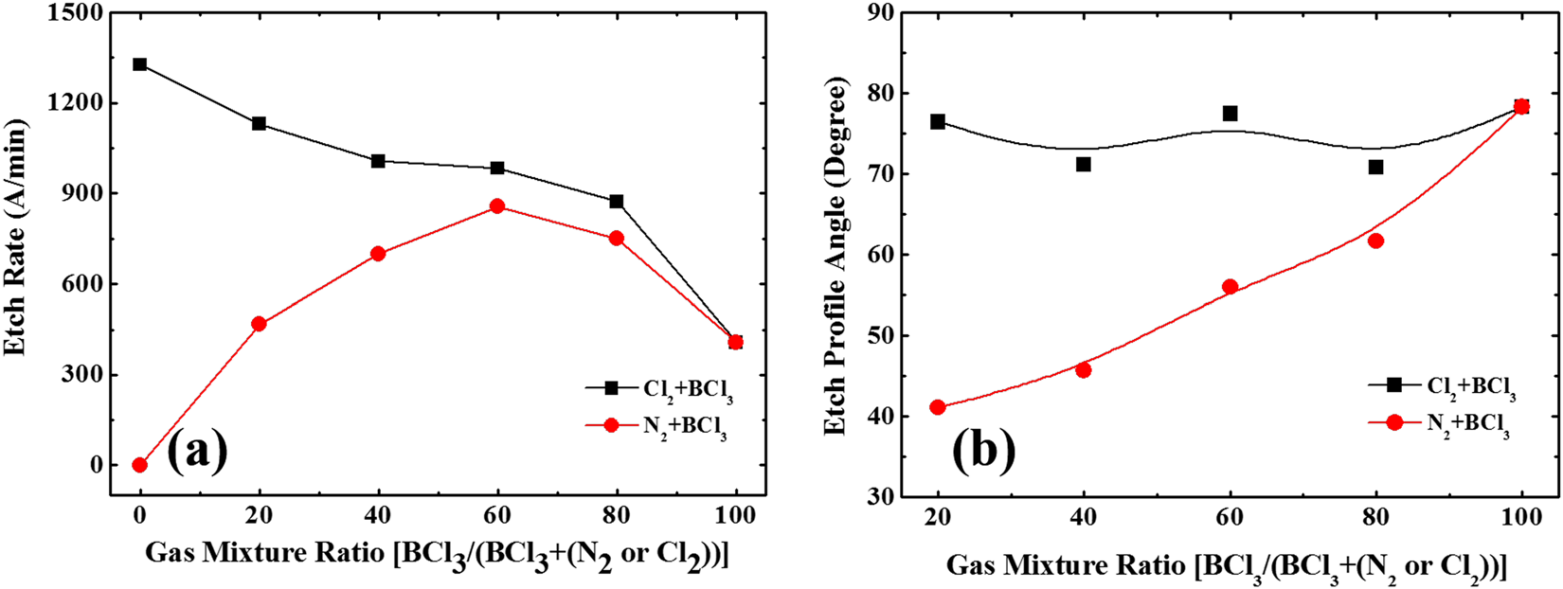
Etching rate and etching profile angle for SiC etching under different gas mixture ratios. (**a**) Etching rate using BCl_3_ mixed with Cl_2_ (black line) and with N_2_ (red line), demonstrating the high etching rate characteristics of BCl_3_ + Cl_2_ gas mixtures. (**b**) The BCl_3_ + N_2_ gas mixture (red line) achieves controllable etching profile angles between 40**°** and 80**°**, varying almost linearly with the mixture ratio. In contrast, unstable characteristic is observed for the other gas mixture, BCl_3_ + Cl_2_ (black line).

Given this process, a BCl_3_-based etching atmosphere is considered for bevel SiC etching. Figure 3(a) demonstrates the SiC etching rate changes according to the contents of the processing gas; as the total flow rate of the processing gas is kept constant at 40 sccm, the content ratio of the N_2_ or Cl_2_ gas is adjusted to obtain various ratios (from 0 to 100%) of the whole gas mixture. During the BCl_3_ + Cl_2_ gas mixing process, the etch rate is observed to be inversely proportional to the content of BCl_3_. This occurs because the addition of chloride gas reduces the densities of positive ions and electrons. As a consequence of this higher dissociation threshold energy, BCl_3_ can absorb more energy before molecular dissociation than Cl_2_^32^. The highest SiC removal rate (1330 Å/min) is observed when the BCl_3_ + Cl_2_ gas mixture is held with a BCl_3_ content ratio of 0%. The removal rate decreases dramatically to 407 Å/min at a BCl_3_ content ratio of 100%.

In contrast, the SiC etching rate when using a BCl_3_ + N_2_ gas mixture does not vary consistently with BCl_3_ ratio. Instead, the etching rate increases almost linearly as the BCl_3_ content ratio increases from 0 to 60%, but decreases significantly and abruptly beyond this point. The highest removal rate (860 Å/min) was observed at a 60% content ratio, a rate which falls to 407 Å/min as the BCl_3_ content ratio reaches 100%. The addition of BCl_3_ to N_2_ plasma initially results in a significant increase in etching rate, caused by increased BCl_3_ dissociation. The dissociation of BCl_3_ stems from an increase in electron temperature, the result of energy transferred from the N_2_ metastable. As such, the N_2_ metastable is responsible for the both the increased dissociation and enhanced production of the etch species. However, the continuous increase in BCl_3_ content ends up reducing the etching rate, which is most likely the result of a decrease in the effective volume density of the BCl_3_-ions and reactive Cl^−^. Such a decrease reduces the ion bombardment and associated chemical reaction between the SiC substrate and Cl^−^-atoms while the ICP power is maintained; thereby reducing the number of reactive species and ultimately leading to a decrease in etching rate.

In accordance with previously reported quadrupole mass spectrometry results^33^, BCl_2_, BCl, Cl_2_, and Cl will exist as a function of the percentage of N_2_ in the flow. The Cl_2_ intensity can increase up to several times the initial value for BCl_3_ in the presence of 60% N_2_. Under these conditions, the Cl^−^ intensity will decrease, but continues to exhibit a local maximum at 80%. The recombination tendency of Cl^−^, reforming Cl_2_ during transport to the mass spectrometer, may account for the low measurements Cl^−^ intensity that result in the observed high SiC etching rate.

Figure 3(b) shows the obtained etching profile angle as a function of the gas mixture ratio. Large etching angles—ranging from 70**°** to 77**°**—are achieved by applying the processing gas mixtures BCl_3_ + Cl_2_, whereas in the case of BCl_3_ + N_2_ the angle tends to increase with the gas mixture ratio. An etching profile angle of approximately 40**°** is noted at a BCl_3_ + N_2_ gas mixture ratio of 20%, and it increases to approximately 75**°** at a BCl_3_ gas mixture ratio of 100%. However, the use of a BCl_3_ + Cl_2_ gas mixture plasma results in (almost perfect) smooth vertical walls with relatively good anisotropy, because of the continual existence of the diboron tetrachloride (B_2_Cl_4_) thin polymer layer, which produces sidewall passivation. We believe that the narrow-range profile angle changes are caused by the generation of chloropolymers on the freshly etched SiC surface, resulting in B_2_Cl_4_ that cannot be easily etched with BCl_3_ and/or Cl_2_. The controllable angle characteristics of BCl_3_ + N_2_ gas mixtures can be attributed to the results of anisotropic etching and N_2_-promoted passivation. First of all, the mask edges might be oxidized by the pristinely etched SiC surface generated by the O_2_ residual, resulting in SiO_x_ thin films. The oxygen sources can be residual oxygen gases or reaction by-products from the hard mask^34^. As soon as N_2_ (<40%) is added to the BCl_3_ mixture gas, a slight compound-nitride-like passivation layer is deposited. At this point, the SiC sidewall will remain close to vertical, showing the SiO_x_ layer is thick and dense enough to prevent chemical etching reactions between neutral chlorine species and the sidewall. With the continuous increase of N_2_ (>40%), passivation deposition becomes excessive, the etch rate decreases, and the sidewall becomes more extensively profiled. Moreover, a grass-like roughness appears at the bottom of the etched area, caused by the formation of a compound-nitride-like passivation layer, which requires a 1:6 buffered oxide etching process for thorough removal from the thin SiO_x_ film. Similar SiO_x_ and nitride-based passivation layers are observed on InP wafers, etched by BCl_3_/N_2_ and Cl_2_/N_2_ gas mixtures^35, 36^.

**Figure 4:**
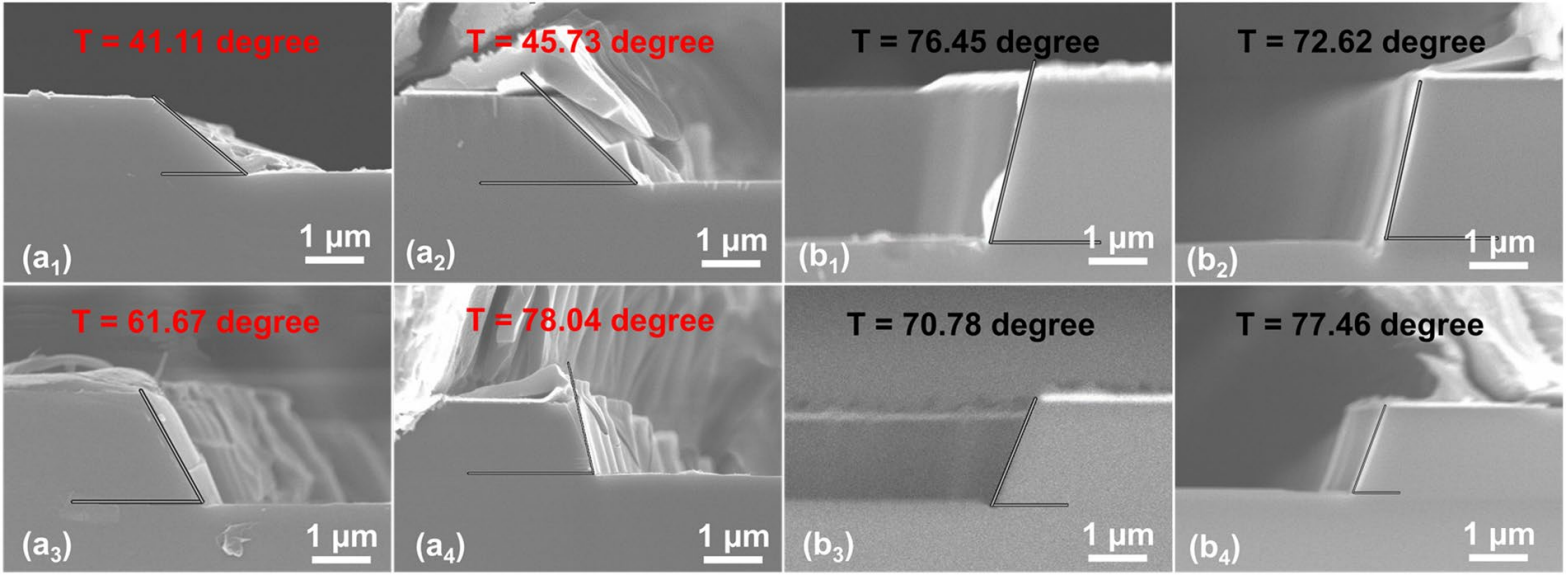
SEM images of the etching profile angles obtained with different mixture ratios of BCl_3_ with (BCl_3_ + N_2_) labelled as (**a**) and (BCl_3_ + Cl_2_) labelled as (**b**), showing that etching angles can be controlled by adjusting the gas mixture ratio. (a_1_) Mixture ratio of 20% results in the lowest etching angle, 41.11°; (a_2_) mixture ratio of 40% results in an etching angle of 45.73°; (a_3_) mixture ratio of 80% results in an etching angle of 61.67°; and (a_4_) BCl_3_ gas alone (100%) results in the largest etching angle of 78.04°. (b_1_) Mixture ratio of 20% results in an etching angle of 76.45°; (b_2_) mixture ratio of 40% results in an etching angle of 72.62°; (b_3_) mixture ratio of 80% results in the lowest etching angle of 70.78°; and (b_4_) BCl_3_ gas alone (100%) results in the largest etching angle of 77.46°.

To investigate the effects of N_2_ and Cl_2_ in a BCl_3_ plasma, gas flow ratio of the two gases are separately varied from 80% to 0%. Under this procedure, an evaluation of improvements in sidewall etching profile control can be conducted, observing the separate effects of N_2_ and Cl_2_ gas. Corresponding SEM images of etching profile angles are shown in Fig. 4(a_1_–a_4_) and Fig. 4(b_1_–b_4_). A wider angle range, from 41° to 78°, is observed when using N_2_ + BCl_3_ as the etching gas mixture; in comparison, using Cl_2_ + BCl_3_ results in larger angles within a more narrow range, from 70° to 77°. These findings show the BCl_3_ + N_2_ mixture is an optimal choice for wide-range, tuneable, large-angle SiC bevel etching.

### Small-angle Bevel Etching

Small-angle bevelled mesa etching demands that focus be placed simultaneously on achieving small angles (~7°) and ensuring surface smoothness, a requirement for effective application in APDs. This prevents the premature breakdown around the mesa edge termination referred to at the beginning of the paper. Aiming for the smallest possible bevelled sidewall increases the depletion width at the surface of the APD, in comparison to bulk. As such, the electric field is lower at the edges than the centre, further contributing to quite high breakdown characteristics and allowing bulk breakdown to precede surface breakdown. In an SF_6_ + O_2_ mixture, the chance of polymer formation during photoresist etching increases greatly with the increase in O_2_ ratio. These polymers, once formed by plasma, function as very tough microetching masks that transfer to the SiC surface and eventually obstruct SiC etching. Additionally, by using SF_6_, more fluoride ions participate in the reaction, creating more by-products. These by-products are then responsible for generating roughness in the polymer surface, because of scattering on the etching surface as a secondary mask^37, 38^. This effect restricts the use of SF_6_ + O_2_ as a gaseous mixture for small-angle bevel etching. BCl_3_ could be another choice for achieving small-angle bevels; however, the anisotropy characteristics of BCl_3_ only allow large-angle bevels (≥70°), unlike some other mixture gases. As mentioned before, if a gas such as N_2_ is added to the BCl_3_ atmosphere, a relatively small-angle bevel (almost as low as 40°) may be formed. However, for the preparation of ultra-small SiC sidewall bevel angles, a larger etching selectivity between the etching mask and the etched object must be obtained, properly adjusted, and controlled.

**Figure 5:**
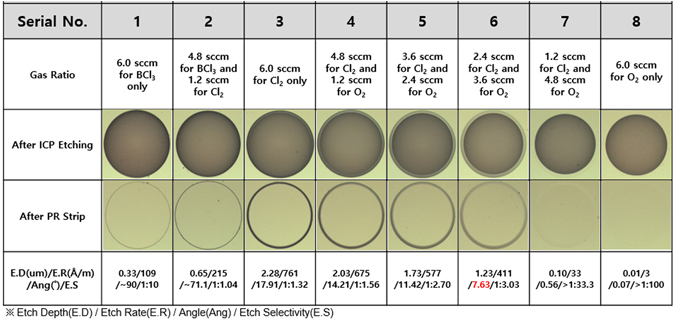
ICP etching profiles for different BCl_3_, BCl_3_ + Cl_2_, Cl_2_, and Cl_2_ + O_2_ gas ratios. As indicated, a high etching depth of 1.23 µm and a low etching profile angle (up to 7.63**°)** can be achieved using Cl_2_ gas (2.4 sccm) in combination with O_2_ gas (3.6 sccm), with a compromised etching rate of 411 Å/min and a selectivity of 1:3.03; 30 min fixed etching time.

**Figure 6:**
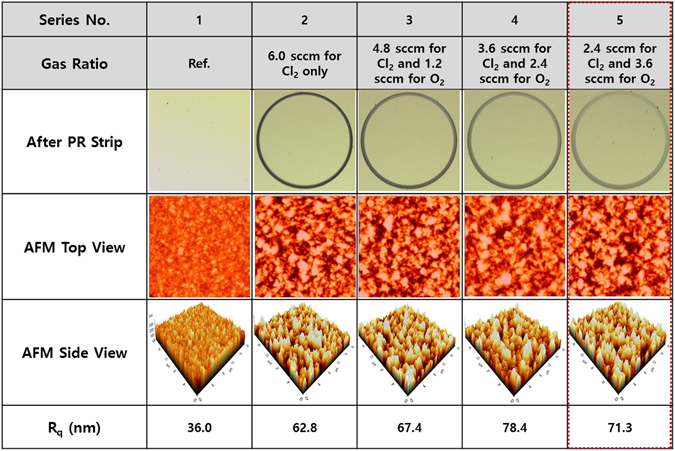
A surface morphological study of the effects of different Cl_2_ and O_2_ ratios, using atomic force microscopy data. The results suggest that on using an appropriate gas mixture ratio, the resultant surface roughness is acceptable for APD applications.

In this study, a well-patterned photoresist is used as the etching mask due to its chemical activity, as shown in Figure S1 and S2 (supplementary section). Given that Cl_2_ is more inert to photoresist than BCl_3_, it enable etching of smaller angles (<20°); and with the addition of a regular photoresist reactant gas (i.e. O_2_), the mixture gas is likely to allow even smaller bevel angles. The smallest bevel etching angle (as low as 7.63°), is achieved by using a Cl_2_/O_2_ gas mixture with a gas flow ratio of 2.4 sccm for Cl_2_ and 3.6 sccm for O_2_ (shown in Fig. 5). This indicates that when the majority of the gaseous mixture is O_2_, photoresist erosion is significantly accelerated without inducing further SiC etching. The etching depth, etching rate, and selectivity thus obtained are 1.23 µm, 411 Å/min, and 1:3.03, respectively. The use of Cl_2_ gas alone at 6 sccm results in a maximum etching depth of 2.28 µm and an etching rate of 711 Å/min, whereas the minimum etching depth of 0.33 µm and an etching rate of 109 Å/min are obtained by using BCl_3_ gas at 6 sccm. The use of Cl_2_ as an etchant results in a small angle, whether alone or mixed with O_2_. As the ratio of O_2_ is increased with respect to Cl_2_ (from 0 to 3.6 sccm of O_2_), the bevel angle is found to decrease from 17.91**°** to 7.63**°**, at the expense of decreasing both the etching depth and etching rate, which decrease from 2.28 to 1.23 µm and from 761 to 411 Å/min, respectively. With the continuous increase of O_2_ from 4.8 sccm to 6.0 sccm, the etch selectivity of all samples increases monotonically with the increasing O_2_ flow rate, which shows that the patterned photoresist is not applicable as a suitable etching mask for SiC ICP-RIE. The surface roughness of the SiC substrate depends on the etching conditions, and therefore remains another important parameter when evaluating the small-angle bevel etching quality, particularly during the APD mesa termination fabrication. The SiC layer surface morphologies produced by different Cl_2_-related gas mixtures are shown in Fig. 6—via AFM—revealing that the use of Cl_2_ + O_2_ gas mixtures causes a slight change of surface morphology. In the absence of SiC etching, surface roughness is found to be 36.0 nm, and increases slightly in the presence of the various Cl_2_ + O_2_ gas mixtures. Using Cl_2_ alone, the surface roughness is found to be 62.8 nm. As the amount of O_2_ increases, the SiC surface roughness deteriorates from 67.4 nm to 78.4 nm. The surface roughness worsens with the formation of oxide films in the presence of excessive O_2_, greatly reducing the etching rate in certain areas^39^. However, when the O_2_ content increases to 3.6 sccm, the surface roughness improves to 71.3 nm. Unevenly etched surfaces occur primarily because of either splits in the metal mask material and plasma polymer residues, which scatter throughout the etching region and form a microscopic mask. Oxygen plasma can help removing this scattered layer. Therefore, adding an appropriate amount of O_2_ can improve the surface roughness.

## Conclusions

In this work, different gas chemistries were used to obtain a variety of bevel angles for SiC substrates, and their performance for ICP-RIE etching was investigated and analysed. Results showed that a high etching rate and improved selectivity for vertical etching can be obtained with the use of a fluorine-based gas mixture (i.e. SF_6_) in conjunction with different ratios of O_2_ and an Ni mask. Additionally, it was found that a wide, controllable range of bevel angles can be achieved in large-angle bevel structure formation by using a BCl_3_ + N_2_ gas mixture. It was also demonstrated that Cl_2_ gas can effectively achieve small-angle bevel structures, and that the addition of different ratios of O_2_ can further reduce the small-angle bevel angle to as low as 7**°**. Furthermore, it was shown that surface morphology is not significantly affected by the use of Cl_2_ and O_2_ etching gas mixtures, with a smooth surface being maintained on the SiC layer.

The vertical SiC etching process developed here can be applied to large volume manufacturing for future MMIC backside via etching and source grounding applications. Large-angle bevels in SiC etching are significant for the bevel mesa of diodes, transistors, and switches. Moreover, the issue caused by electric field concentration could be overcome through large-angle bevel structure, so that high performance and high reliability can be obtained for the devices including PIN diodes, Schottky diodes, SITs, and JFETs. Finally, with optimal small-angle SiC bevel structures and a smooth surface morphology, the leakage current of the APDs could be effectively reduced, and greatly improved breakdown properties will be achieved as predicted in other works.

## Electronic supplementary material

Supplementary information
