# Supplementary material for: Vertical and bevel-structured SiC etching techniques incorporating different gas mixture plasmas for various microelectronic applications
Source: Sci Rep. 2017 Jun 20;7:3915. doi: 10.1038/s41598-017-04389-y (PMC5478634; doi:10.1038/s41598-017-04389-y)
Supplement: Supplementary file 1 — Supplementary information [file 41598_2017_4389_MOESM1_ESM.pdf]

Supplementary Information

Vertical and bevel‒structured SiC etching techniques incorporating different gas mixture plasmas for various microelectronic applications

Ho-Kun Sung [3,#], Tian Qiang[2,#], Zhao Yao[4,*], Yang Li[5], Qun Wu[1], Hee-Kwan Lee[3], Bum-Doo Park[3], Woong-Sun Lim[3], Kyung-Ho Park[3] and Cong Wang[1,2,*]

[1]*Department of Microwave Engineering, Harbin Institute of Technology, Harbin 150001, China*
[2]*Department of Electronic Engineering, Kwangwoon University, 20 Gwangun-Ro, Nowon-gu, Seoul 139701, Republic of Korea*
[3]*Korea Advanced Nano Fab Center (KANC), 109 Gwanggyo-Ro, Yeongtong-gu, Suwon-si, Gyeonggi-do, 443-270, Republic of Korea*
[4]*College of Electronic and Information Engineering, Qingdao University, Qingdao 266071, China*
[5]*School of Information Science and Engineering, University of Jinan, Jinan 250022, China*
[#]*These authors contributed equally to this work.* [*]*Correspondence and requests for materials should be addressed to Zhao Yao (email: yao9074@hotmail.com) or Cong Wang (email: kevinhunter0414@hotmail.com)*

1. Formation of Small-angle Bevel SiC Structure

The used lithography for the etching of a bevelled mesa APD employs a 6-µm-thick AZ4620 photoresist mask spun at 3000 rpm for 40 s in the track, and then baked for 180 s at 100 °C, after which a photolithographic process is performed with 400 mJ/cm^2 of exposure energy. Following exposure, the AZ4620 is then developed in AZ300MIF developer for 280 s, followed by a hotplate bake at 140 °C for 300 s to pull in the edges of the photoresist, thus creating a rounded dome profile [as shown in Fig. S1(a)]. According to Yan *et al.*, the bevel angle of the photoresist decreases with both higher bake temperatures and longer bake times. This is because the higher and longer bake temperatures increase the surface tension, thereby reducing the bevel angle of the photoresist. An important aspect of mesa formation is the control of the mesa bevel angle. The samples are then etched using different gas

mixtures at a total 6.0 sccm flow rate, 200 W chunk power, 3 mTorr chunk pressure, during 35 min [as represented in Fig. S1(b)]. During ICP-RIE, the photoresist is etched at a faster etching rate than the SiC surface; therefore, the SiC mesa acquires the (exaggerated) angle β of the photoresist shown in Fig. S1(c), which is given by:

$$\tan\beta = \left(\frac{R_{SiC}}{R_{Photoresist}}\right)\cdot\tan\alpha \approx \tan^{-1}\left(\frac{2T}{D}\times\frac{R_{SiC}}{R_{Photoresist}}\right) \quad (1)$$

where α is the bevel angle of the photoresist before etching, R_{SiC} and $R_{Photoresist}$ are the SiC and photoresist etching rates, respectively, $R_{SiC}/R_{Photoresist}$ is the selectivity ratio of the etched SiC to the etched photoresist mask, D is the mesa diameter defined by the mask, and T is the centre thickness of the photoresist. Figure S1(d) shows the resulting bevelled mesa formation on SiC after removal of the residual photoresist. Equation (1) shows that, to obtain a small bevel angle β, small $R_{SiC}/R_{Photoresist}$ ratios or small values of α should be used. For a given α, a feasible approach to tune the bevel angle is to vary the $R_{SiC}/R_{Photoresist}$ ratio.

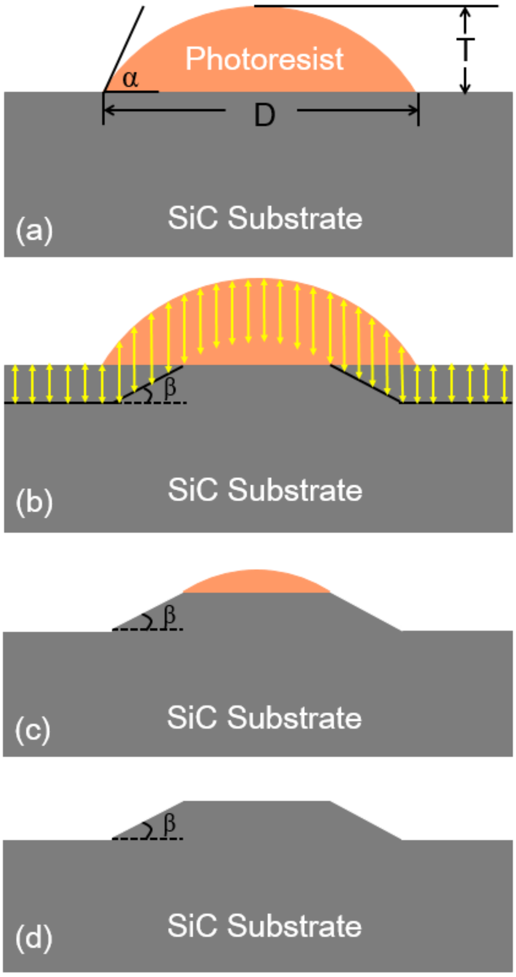

Figure S1. Small-angle bevel etching process illustration. (**a**) SiC substrate with well-patterned dome-typed photoresist. (**b**) ICP-RIE etching depth represented by yellow arrows on the SiC substrate and photoresist. (**c**) After removal by the ICP-RIE, with remaining photoresist. (**d**) After removing the residual photoresist, thus completing the SiC small-angle bevelled mesa edge fabrication.

2. Small-angle Bevel SiC APD Mesa Termination Fabrication Process

Scanning electron microscopy images of the optimized Cl_2 + O_2 gas mixture—2.4 sccm Cl_2 and 3.6 sccm O_2—are shown in Fig. S2, which represent all the significant states of the small-angle bevel APD mesa termination fabrication process: before photoresist baking [Fig. S2(a)], after the photoresist reflow process [Fig. S2(b)], after the proposed Cl_2/O_2 ICP-RIE etching [Fig. S2(c)], after applying the photoresist strip [Fig. S2 (d)], and finally after obtaining the required small-angle bevel etched profile angle. The top view of the obtained mesa in Fig. S2(e) reveals the design consideration for patterning: an inner diameter of 190 µm and a slant height of 12 µm. Based on the measured data, we did successfully obtain a diameter of 191 µm and a slant height of 11.9 µm. Figure S2(f) illustrates an enlarged cross-sectional view of the small-angle bevel-etched SiC surface, which forms an angle of 7.63° with the base of the SiC layer.

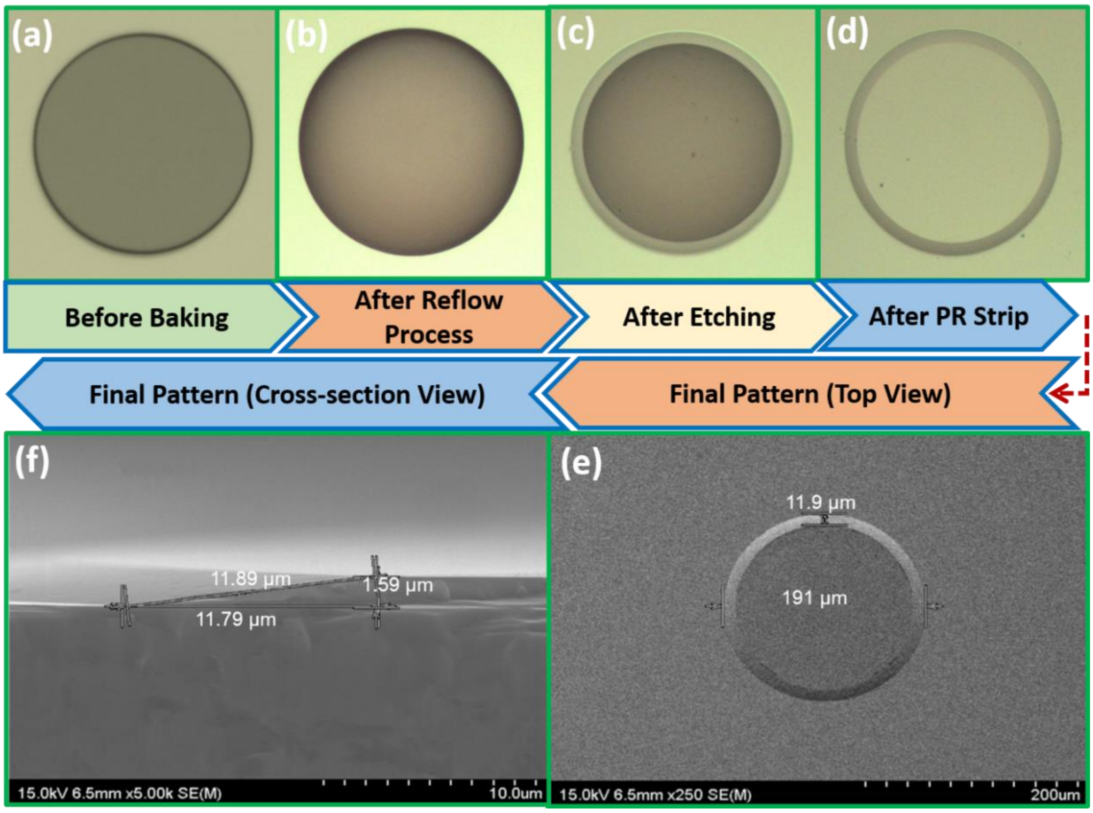

Figure S2. Most significant states of the small-angle bevel SiC APD mesa termination fabrication process. (**a**) Before photoresist baking. (**b**) After the photoresist reflow process. (**c**) After the Cl_2/O_2 ICP-RIE process. (**d**) After applying the photoresist strip. (**e**) Top view (SEM image) of a mesa with a low etching profile angle (7.63°), obtained with the optimized gas

mixture of Cl_2 (2.4 sccm) and O_2 (3.6 sccm). (**f**) Enlarged cross-sectional view (SEM image) of the obtained small-angle bevel-etched SiC substrate.
